# BoNT/A in the Urinary Bladder—More to the Story than Silencing of Cholinergic Nerves

**DOI:** 10.3390/toxins14010053

**Published:** 2022-01-12

**Authors:** Hodan Ibrahim, Jacquie Maignel, Fraser Hornby, Donna Daly, Matthew Beard

**Affiliations:** 1 Department of Pharmacy and Biomedical Science, University of Central Lancashire, Preston PR1 2HE, UK; HIbrahim1@uclan.ac.uk (H.I.); DDaly3@uclan.ac.uk (D.D.); 2 Ipsen Innovation, 5 Avenue du Canada, 91940 Les Ulis, France; jacquie.maignel@ipsen.com; 3 Ipsen Bioinnovation, 102 Park Drive, Milton Park, Abingdon OX14 4RY, UK; fraser.hornby@ipsen.com

**Keywords:** botulinum neurotoxin, neurotoxin, bladder, urology, urothelium, SNAP-25, SV2

## Abstract

Botulinum neurotoxin (BoNT/A) is an FDA and NICE approved second-line treatment for overactive bladder (OAB) in patients either not responsive or intolerant to anti-cholinergic drugs. BoNT/A acts to weaken muscle contraction by blocking release of the neurotransmitter acetyl choline (ACh) at neuromuscular junctions. However, this biological activity does not easily explain all the observed effects in clinical and non-clinical studies. There are also conflicting reports of expression of the BoNT/A protein receptor, SV2, and intracellular target protein, SNAP-25, in the urothelium and bladder. This review presents the current evidence of BoNT/A’s effect on bladder sensation, potential mechanisms by which it might exert these effects and discusses recent advances in understanding the action of BoNT in bladder tissue.

## 1. Botulinum Neurotoxins

BoNT/A is a potent toxin with zinc metalloprotease activity produced by *Clostridium botulinum*. There are at least six other serotypes of BoNTs which have been named BoNT/A to /G, with over 40 different subtypes [1]. BoNT/A is currently approved for the treatment of a variety of medical ailments including cervical dystonia, strabismus and migraine (FDA) [2]. BoNTs inhibit exocytosis by targeting and cleaving soluble N-ethylmaleimide sensitive factor attachment protein receptor (SNARE) proteins, which are essential for vesicular release from cells. This is achieved in a three-step process: (1) binding to its receptor, (2) translocation into the cell, and (3) proteolysis (of the SNARE).

BoNT/A receptor binding comprises a dual interaction between ganglioside and protein receptors. The protein receptor is called SV2, which becomes exposed on the surface at higher levels in neurons undergoing high levels of exocytosis. Binding to this receptor allows BoNT/A to target the most active neurons preferentially [3]. Once bound, BoNT is endocytosed into a synaptic vesicle compartment, and as the pH is lowered within the vesicle during acidification, a specialized domain within the BoNT (called Hn) produces a pore in the vesicle membrane to allow the light chain to cross into the cytosol [4]. The liberation of the light chain into the nerve terminal cytosol allows it to cleave its SNARE target, which is SNAP-25 for BoNT/A.

**Figure 1:**
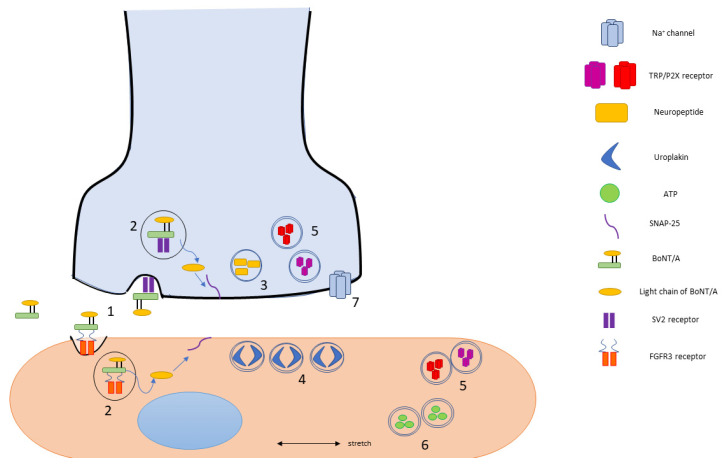
Proposed mechanisms of BoNT/A entry and action in the modulation of bladder sensation based on the current literature. (**1**) The BoNT/A molecule enters cells by receptor-dependent uptake using the well-characterized SV2 receptor and possibly the more recently described FGFR3 receptor. (**2**) After endocytosis, the BoNT/A light chain is transcytosed into the cytoplasm and cleaves SNAP-25, its SNARE target. SNAP-25 cleavage has potential to disrupt a number of sensory signaling pathways which depend on vesicular release. Such pathways include (**3**) neuropeptide release; (**4**) uroplakin protein facilitation of surface area expansion during bladder filling; (**5**) receptor localization in plasma membrane, such as TRP or P2X receptors; (**6**) stretch induced vesicular release of ATP; and (**7**) Na^+^ channel excitability in nerve terminal membranes.

SNAP-25 cleavage inhibits neuronal exocytosis, which leads to muscle paralysis after intoxication of motor neurons [5]. However, there is evidence that the action of BoNT/A is not restricted to muscle paralysis, as patients report reduced sensory symptoms post BoNT/A injection. Animal studies have shown direct inhibition of sensory nerves such as the afferent nerves of the meninges that mediate migraine headaches [6] and the urinary bladder [7]. These data support a direct effect of BoNT/A on afferent nerves, and the purpose of this review is to further investigate evidence supporting this activity and the mechanisms behind the observed phenomenon, summarized in Figure 1.

## 2. Effects of BoNT/A in Bladder Disorders

Clinical and non-clinical observations of BoNT/A effects in bladder tissue are increasingly difficult to reconcile with a simple drug mechanism consisting only of inhibiting muscle contraction by blocking acetylcholine (ACh) release at neuromuscular junctions. Patients with overactive bladder who receive intravesical (within the bladder) injection of BoNT/A report sensory effects such as reductions in urgency and pain during the bladder filling phase [8,9,10]. During this phase, the detrusor muscle is relaxed and sympathetic nervous stimulation predominates [11]. Similar sensory effects are also reported after resiniferotoxin administration, which is a TRPV1 specific toxin that acts directly on sensory nerves [12,13]. Animal studies have shown direct inhibition of sensory nerve activation in the urinary bladder [7,14]. Together, these observations suggest BoNT/A may act to reduce afferent sensory nerve activation in bladder tissue. The purpose of this review is to examine in detail the evidence leading to this proposal and potential molecular mechanisms by which BoNT/A might act to inhibit generation of action potentials by sensory neurons.

Overactive bladder (OAB) is a lower urinary tract (LUT) syndrome characterized by urgency, which is defined as a strong, unavoidable urge to urinate that cannot be deferred, which may be accompanied by incontinence [15]. Other symptoms include frequency, which means having to use the bathroom more often, and nocturia, or sleep disturbances caused by increased nightly bathroom visits [15]. Interstitial cystitis/painful bladder syndrome (IC/PBS) is a chronic LUT disorder in which patients also experience frequency and nocturia, however IC/PBS patients suffer from discomfort or pain while OAB patients largely do not [15,16]. The pain is associated with bladder filling, as the volume of urine increases the sensation of discomfort rises concomitantly, so the patient has to urinate for fear of increasing pain, whereas OAB patients sense filling without pain and the main motivation to urinate is the threat of leakage [17]. The etiologies of LUT disorders such as OAB and IC/PBS have not been characterized fully, however several theories have been developed which implicate the different cell types in the bladder wall, as well as disruptions to the neural control mechanisms in the pathophysiology. Unfortunately, there are currently no cures for LUT disorders, and treatments include oral medications which are relatively ineffective and have common and frequently intolerable side effects [2,18,19].

## 3. Bladder Physiology

As the bladder fills with urine, the detrusor smooth muscle of the bladder wall relaxes to accommodate the increasing volume without causing a significant rise in intraluminal pressure. Filling is detected by the urothelium and mechanosensory afferent nerves which project into the urothelium, the suburothelial layer and the detrusor smooth muscle [20]. At lower volumes, the sympathetic pathway is activated to facilitate urine storage. In this storage phase, noradrenaline is released from local sympathetic nerve endings and causes relaxation of the detrusor smooth muscle through activation of β3 adrenoreceptors, and the concomitant contraction of the urethral sphincter through activation of α adrenoreceptors [21,22]. To initiate voiding of the bladder, the parasympathetic postganglionic nerves release acetylcholine (ACh), which activates muscarinic M3 receptors on the smooth muscle to induce contraction. Concurrently the urethral sphincter is relaxed by nitric oxide and micturition is initiated [23,24].

Up until the late 1990s, we believed the inner epithelial lining of the bladder, a structure termed ‘the urothelium’, was nothing more than a simple barrier between urine and blood. However, conclusive evidence over the last two decades has shown that the urothelium is involved in bladder sensation.

Much of the work on the bladder urothelium has been conducted using tissues from rodents or rodent models which have a greater purinergic contribution to function than normally found in the human bladder [25]. While ATP signaling is undoubtedly the most well characterized urothelial pathway, there is also now a comprehensive body of literature which shows that in addition to ATP, the urothelium has the capacity to release a host of mediators including ACh [26,27], nitric oxide [28], and substance P [29]. These neurotransmitters can initiate signaling cascades to stimulate or inhibit the urothelial cells themselves, afferent nerves and/or smooth muscle cells. The purinergic hypothesis describing ATP as a neurotransmitter was developed by Burnstock in the 1970s and confirmed in the late 1990s by Ferguson et al. (1997) who showed that the bladder urothelium detects mechanical stretch and releases ATP in response [30,31,32,33,34,35]. Later, Vlaskovska et al. (2001) showed that mice deficient in the purinergic P2X3 receptor exhibited reduced afferent nerve responses to bladder distension compared to wild type mice [36]. Rong et al. (2002) showed distension-induced afferent firing was amplified by P2X3 agonists and inhibited by P2X3 antagonists [37]. Taken together, this has led to our current understanding that ATP is released from the urothelium in response to filling of the bladder, and acts on the urothelial cells themselves to potentiate membrane traffic and accommodate increasing stretch, as well as downstream on underlying sensory nerve terminals to drive afferent transmission during bladder filling.

Nitric oxide (NO) is a gaseous neurotransmitter that has been implicated in driving the inhibitory limb of bladder sensation [28,38]. In this way, ATP and NO have been hypothesized to act antagonistically to modulate bladder sensation, and disruptions in their release could be behind manifestations of overactive and underactive bladder [39]. Interestingly, Smith et al. (2008) found that ATP and NO release was dysregulated in a rat model of chronic spinal cord injury, with significantly increased ATP release and significantly decreased NO release resulting in bladder hyperactivity [40]. This disruption of ATP and NO release was normalized by BoNT/A treatment [40]. The modulation of stretch induced ATP and NO release by BoNT/A has been shown by multiple groups [7,40,41,42,43] and raises important questions about its action on bladder sensation.

## 4. Use of BoNT/A to Treat Bladder Disorders

Current UK National Institute for Health and Care Excellence (NICE) guidance recommends 100 Botox unit (100 U) injections of BoNT/A in the bladders of patients with OAB, increasing the dose to 200 U if symptoms persist [19]. Table 1 outlines the most common bladder disorders, and their abbreviations as defined by the International Continence Society [15]. Dykstra et al. (1988) were the first to inject BoNT/A into rhabdosphincter of patients with spinal cord injury and detrusor-sphincter dyssynergia, finding that the majority of the patients had improvements in urinary retention [44]. Over the years, other potential urological applications were tested, and BoNT/A was approved for use in the US for OAB in patients not responsive or intolerant to anti-cholinergic drugs [2]. Schurch et al. (2000) were the first to inject BoNT/A directly into the bladder wall, using a cystoscope to enter the bladder through the urethra [45]. Since the turn of the millennium, there have been a number of clinical trials studying the effectiveness of BoNT/A in treating OAB using protocols that vary in total dose, areas of injection, and numbers of injections [8,9,46,47,48,49]. Denys et al. (2012) reported that injection of 150 U BoNT/A led to significant improvements in urodynamic parameters including voided volume, volume at first contraction and volume at strong desire to void [46]. Chapple et al. (2013) reported a decrease in episodes of incontinence and urgency incontinence of 52% and 53%, respectively, two weeks after treatment with 100 U BoNT/A [8].

The potential use of BoNT/A for IC/PBS was first explored by Smith et al. (2004), who found frequency, nocturia, and pain were significantly reduced post-treatment [10]. Their injection protocol included 200 U of Botox^®^ and 200 U Dysport^®^ into the trigone, leading to reduction of IC/PBS symptoms including frequency and pain. These findings were replicated by Giannantoni et al. (2006), Kuo et al. (2009), and Pinto et al. (2010) who have all shown intravesical toxin injections reduced symptoms of IC/BPS [50,51,52]. A double blind, placebo-controlled clinical trial conducted by Kuo et al. (2016) concluded that patients receiving suburothelial injections of 100 U Botox^®^ exhibited significantly less pain and higher bladder capacity than the normal saline group [9]. Although botulinum toxins have not been approved for use in IC/PBS patients, the current evidence suggests that patients in these clinical trials reported relief from pain and improved bladder function post-treatment.

The benefits of BoNT/A may be due to non-classical mechanisms affecting sensation from the bladder. Many clinical trials have shown significant reductions in the sensation of urgency in OAB patients [8,48,53] and pain in IC/PBS patients [50,54]. BoNT/A treatment significantly improves patients’ quality of life [51]; boosts in quality-of-life scores were found to be significantly correlated with reductions in episodes of urgency and incontinence [55]. Surprisingly despite its clinical use, the mechanism which underlies the effectiveness of BoNT/A for the treatment of LUT disorders has not been fully described. BoNT/A could be working via a well-established mechanistic pathway involving the paralysis of the bladder smooth muscle by silencing cholinergic nerves through cleavage of SNAP-25 [56]. The appearance of urinary retention in those treated with BoNT/A certainly lends weight to this idea, the weakening of the detrusor muscle via the inhibition of ACh signaling needed to induce bladder contraction. This evidence suggests BoNT/A could be influencing both afferent and efferent limbs of neuronal signaling in the bladder to generate the effects reported by patients.

## 5. Effect of BoNT/A on Sensory Nerves—Direct Action

Bladder afferent nerves are composed of C- and Aδ-fibers. Aδ-fibers have been shown to be responsible for contributing to normal micturition through transducing non-painful, physiological stretch [57], whereas C-fibers seem to have high thresholds and transduce painful stimuli caused by supra-physiological stretch at pressures above 30 mm Hg [58], or they are unresponsive to mechanical stimuli and only start responding after sensitization by chemical irritants or inflammation [58]. Collins et al. (2013) found that BoNT/A attenuated afferent nerve responses to bladder filling, reducing responses from both low threshold (69% decrease) and high threshold (65% decrease) afferents, however they did not measure conduction velocity directly in this study [7].

BoNT/A attenuation of nerve response has also been observed in sensory nerves in other tissue types, for example, the meningeal nerves that mediate migraine pain. Burstein et al. (2014) conducted a study on the effect of BoNT/A application on mechanosensitivity of rat meningeal afferent nerves by recording afferent nerve responses to stimulation by von Frey filaments with increasing force, while measuring conduction velocity to identify nerve fibres as C- and Aδ-fibers [6]. In this study, BoNT/A reduced the firing of C-fibers, which are responsible for the transmissions of pain signals to the CNS, suggesting that BoNT/A can directly alter the transmission of sensory nerve signals, at least in this tissue [6]. Interestingly, BoNT/A did not affect firing of Aδ-fibers, which suggests BoNT/A’s effects on the meningeal afferents are most prominent in supraphysiological settings, thus the authors propose BoNT/A reduces the SNARE dependent expression of C-fiber-specific ion channels, such as TRPA1, TRPV1, and P2X3, which has been shown both in vitro and clinically [6,59,60]. Interestingly, there is also a possibility BoNT/A could directly inhibit afferent nerve excitability by inhibiting ion channel activation, as Shin et al. (2011) reported BoNT/A inhibited tetrodotoxin (TTX)-sensitive and TTX-resistant sodium currents in DRG neurons [61]. This finding deserves further study and could provide important insights to SNAP-25 independent effects of BoNT/A.

It is, however, also important to note that these studies were conducted in isolated rodent tissues and may not directly translate to human tissue. Any potential effect on human sensory nerves in bladder could be affected by tissue and species differences between the model systems and human bladder, such as depth of penetration of BoNT/A into the bladder wall to reach the nerve terminal. For example, the mouse urothelium is composed of two or three layers of cells on top of each other, while human urothelium has up to six layers [62,63]. Krhut et al. (2010) performed a pilot study intravesically instilling BoNT/A into bladders of patients with OAB, finding reductions in episodes of incontinence and increased bladder volumes [64]. However, this effect lasted around six weeks, while the effects of injected BoNT/A have been reported to persist up to six months [46,47]. This may suggest that the duration of BoNT/A effect is affected by the degree of penetrance into the bladder, as injection inserts it deeper into the bladder wall, it could exert its effects in more cell types. Some studies have used protamine sulfate, a compound that has been shown to degrade the urothelium, before BoNT/A treatment to increase penetrance and reach the suburothelial fibers directly [65,66]. Chuang et al. (2004) found reduced pain responses to acetic acid after intravesical instillation of protamine sulfate and BoNT/A in rats compared to protamine sulfate and saline [65]. This suggests that increasing penetrance of BoNT/A could facilitate its activity in silencing afferent nerves.

One interesting suggestion is that BoNT/A could work by inhibiting the insertion of sensory receptors into the neuronal membranes, as SNAP-25 is necessary for the insertion of TRPV1 into the neuronal plasma membrane [67]. In a landmark study with over 500 citations, Apostolidis et al. (2005) described a significant reduction in TRPV1 and P2X3 immunoreactivity in bladder suburothelial nerve fibres after BoNT/A injection in patients with detrusor overactivity [59]. More evidence for this idea has also been provided by preclinical studies in a variety of tissue types, as a significant reduction of TRPV1 and TRPA1 expression in the neuronal membrane of trigeminal nerves [60,67,68], DRG neurons [69,70,71,72] as well as rat urothelium [73] has been observed. As those receptors have been shown to mediate mechanosensation in visceral afferent nerves in preclinical studies [74,75,76,77], it is plausible that BoNT/A could ameliorate pain not only by inhibiting the release of nociceptive neurotransmitters [78], but also downregulating the receptors they activate.

## 6. Effect of BoNT/A on Sensory Nerves—Changes in Neuropeptide Release

BoNT/A-mediated cleavage of SNAP-25 has been reported to lead to the inhibition of neuropeptide release. In the porcine DRG, substance P, and CGRP immunoreactivity was significantly reduced following intramural injection of BoNT/A [78]. In in vitro cultures of DRG neurons, Welch et al. (2000) reported dose dependent cleavage of SNAP-25 as well as inhibition of potassium evoked release of substance P [79]. Interestingly, Purkiss et al. (2000) also found BoNT/A inhibited potassium evoked substance P release from DRG neurons, but not high concentration capsaicin evoked release. High-concentration capsaicin was able to induce substance P release even in a Ca^2+^-depleted system. As SNAP-25-mediated vesicular release is Ca^2+^-dependent, the authors suggested the capsaicin evoked release may have been through a non-SNAP-25-mediated mechanism [80].

Nerve Growth Factor (NGF) is a peptide that can modulate bladder sensory nerve activity, and its overexpression is implicated in the development of OAB and IC/PBS [81,82,83,84]. Liu et al. (2009) reported higher urinary NGF levels in bladders of patients with idiopathic detrusor overactivity (IDO), which was significantly reduced after BoNT/A injections into the detrusor [85]. The mechanism underlying reduced NGF release from the bladder after BoNT/A treatment could be due to inhibition of exocytosis, as it is stored and released through vesicular mechanisms [86,87]. Interestingly, it has been shown in a preclinical study that intradetrusor injection of BoNT/A reduced expression of NGF in the urothelium and detrusor [73]. The mechanism behind this reduction in expression is unknown, BoNT/A could directly interfere with the pathway responsible for NGF transcription, or downregulation could be the result of an indirect effect. Inhibition of SNARE-mediated exocytosis could lead to a build-up of NGF containing vesicles in the cell, which could affect transcriptional regulation. As NGF is associated with the development of urological disorders, reduced NGF expression and release could be part of BoNT/A mediated alleviation of symptoms of OAB patients.

## 7. Effect of BoNT/A on Sensory Neurotransmission—Changes to Urothelial Function

BoNT/A could be exerting an action on the sensory nerves via an indirect mechanism involving the bladder urothelium. Neuron-like properties of the urothelium were identified after a series of studies discovered the release of neurotransmitters from the urothelium [26,27,30], as well as expression of various receptors for those neurotransmitters [88,89,90,91,92]. Contrary to neuronal ACh release, which occurs through SNAP-25 mediated exocytosis, ACh release from the urothelium is mediated by organic cationic transporters [93]. Urothelial cells do not express the vesicular acetylcholine transporter (VAChT) [93]. In contrast, at least some portion of ATP release from the urothelium appears to be vesicular [94]. Nakagomi et al. (2016) conducted a study on vesicular nucleotide transporter (VNUT), a recently described transporter shown to be responsible for vesicular release of ATP, to show its effect on ATP release in the bladder. They found that VNUT^−/−^ primary urothelial cells exhibited significantly reduced ATP release in response to weak stretching (10% stretch) but not strong stretching (20% stretch) [33]. They also conducted experiments applying BoNT/A to wild type urothelial cells prior to stretching and found the same pattern of response, BoNT/A significantly inhibiting the weak stretch but not strong stretch induced ATP release [33]. Collins et al. (2013) also found reduced intraluminal ATP release in response to bladder distension after BoNT/A treatment [7]. These results suggest BoNT/A acts directly on urothelial cells to inhibit vesicular release of ATP, and strong stretch initiates different ATP release pathways that are SNAP-25 independent, such as connexin and pannexin channels.

In addition to reducing excitatory transmission, there has been evidence pointing to BoNT/A upregulating inhibitory transmission, through increased production of nitric oxide (NO), although the mechanisms behind these phenomena are unclear. Smith et al. (2008) and Collins et al. (2013) suggested BoNT/A alters sensory signaling by disrupting the excitatory/inhibitory pathways of the urothelium and afferent nerves; however, this idea has not received much attention since publication [7,40]. Aizawa et al. (2011) found inhibition of afferent nerve firing following modulation of the NO pathway. By instilling rat bladders with NO precursor L-arginine and finding reductions in afferent excitation in response to bladder filling, while NO synthase inhibitor L-NAME increased responses to filling [95]. This suggests that if BoNT/A treatment leads to an increase in NO, [96,97] it could result in reduced afferent nerve responses to distension. Although these findings are fascinating, it is quite unclear how BoNT/A could affect NO production, as the production of NO is mediated by eNOS, which is not regulated by SNAP-25 or other SNAREs. Further study in this area is necessary and could potentially provide more indications by which BoNT/A could be used as treatment.

BoNT/A has been shown to inhibit stretch mediated ATP release from the urothelium [7,40,41,42,43]; however, it is not clear if any other SNARE-mediated intracellular processes of apical umbrella cells are affected [98,99]. ATP released during bladder filling stimulates exocytosis of vesicles containing urothelial plaques, or uroplakins, from the lumen facing umbrella cells, to increase the surface area and accommodate increasing volumes. As the bladder is emptied, the process of compensatory endocytosis is initiated to recover that extra membrane [100]. Whether BoNT/A has an effect on membrane traffic of uroplakin-containing vesicles has not been studied directly; potentially, this could have downstream effects on transduction of mechanical stimuli to sensation. The inhibition of ATP release by itself could dysregulate urothelial membrane traffic, Nakagomi et al. (2016) reported reduced exocytosis and less expansion of surface area after bladder filling in urothelial cells of mice that lacked VNUT mediated exocytosis of ATP [33]. This was shown in their Ussing chamber experiments, where VNUT^−/−^ bladders showed significantly lower change in transepithelial resistance as pressure was applied to the mucosal side [33]. Further cell-based studies could improve our understanding of BoNT/A’s effects on bladder mechanosensation.

## 8. SV2 and SNAP-25 Expression in the Urinary Bladder

One potential way to define where BoNT/A is working in the bladder is to localize its protein target. A number of studies have looked at the distribution of SNAP-25 in the bladder, Table 2 shows a summary of these findings. Some studies have been with antibodies specific for cleaved SNAP-25, as a measure of BoNT/A effects, these are summarized in Table 3.

The question of whether SNAP-25 is expressed in the urinary bladder, and in which cell types, is not yet resolved because conflicting data have been reported. Hanna-Mitchell et al. (2015) found that SNAP-25 was present in both human cultured urothelial cells and rat primary urothelial cells through Western blot and immunofluorescence [41]. However, Born et al. (2003) investigated SNARE protein expression in the rat urothelium and did not find SNAP-25 through Western blot and immunofluorescence, concluding that the SNARE complex in the urothelium is composed of SNAP-23, synaptobrevin, and syntaxin [99]. This finding was replicated by Wankel et al. (2016) in mice [98].

Other studies investigating expression of SNAP-25 in the bladder have found it in suburothelial nerve fibers using immunofluorescence techniques [101,102,103]. Coelho et al. (2012) showed by double-labeling cleaved SNAP-25 (cSNAP-25) with vesicular acetylcholine transporter (VAChT), tyrosine hydroxylase (TH), and calcitonin gene-related peptide (CGRP) that 85% of VAChT positive neurons contained cSNAP-25 after BoNT/A exposure, whereas only 42% and 36% of neurons were TH-positive and CGRP-positive, respectively [104]. Interestingly, in the same study Coelho et al. (2012) intravesically instilled BoNT/A and found no cSNAP-25 immunoreactivity in the suburothelial nerve fibers; this suggests that BoNT/A did not intoxicate these cells when instilled into the bladder. These data are in contrast with functional studies that have shown effects of BoNT/A on afferent nerve signaling after instillation into the bladder [7,65,105,106] It is not clear that the functional studies reported alterations in afferent signaling in the suburothelial fibers, it may be that these results were due to effects on different nerve populations.

One possibility is that BoNT/A’s target within rodent bladder isn’t SNAP-25 at all, rather it could be the ubiquitously expressed homologue, SNAP-23, that has been shown in the urothelium [41,98,99]. Although SNAP-25 is BoNT/A’s preferential target, it has been shown to cleave murine SNAP-23 at high concentrations in a proteomic cleavage assay [107] and rat kidney cells [108]. Hanna-Mitchell et al. (2015) reported a decrease in SNAP-23 staining post-BoNT/A treatment in rat urothelial cells [41], which could suggest cleavage. However, as human SNAP-23 has been shown to be resistant to cleavage by BoNT/A [109], species differences may affect translatability of this research. Interestingly, Liu et al. (2015) did not detect cleaved SNAP-25 in OAB patients who responded to Lipotoxin (liposome-encapsulated BoNT/A), although P2X3 expression was significantly decreased in the bladder mucosa [110]. This could suggest BoNT/A working through a non-SNAP-25 mediated mechanism, and further research could provide more insight.

**Table 2:** Summary of studies that have investigated presence of total SNAP-25 within the bladder. ICC—immunocytochemistry, IHC—immunohistochemistry, IF—immunofluorescence, v-SNARE—vesicular SNARE, t-SNARE—target SNARE.

Reference	Species	Where Was SNAP-25 Detected?	Methods	Findings
[41]	Rat and human	Bladder mucosa (urothelium and lamina propria) and primary urothelial cells.	Detected SNAP-25 and SNAP-23 in rat mucosa tissue by performing gel electrophoresis of RNA. Protein expression in human and rat mucosa and primary cells shown by immunoblot and immunocytochemistry.	Human and rat urothelium expresses SNAP-25 and SNAP-23. BoNT/A incubation in rat urothelial cells led to significantly decreased SNAP-25 protein levels, suggesting cleavage.
[101]	Human	Nerve fibers in suburothelium and detrusor, not in urothelial or muscle cells.	Human bladders from organ donors were used for immunofluorescence staining of SNAP-25.	Dense SNAP-25 immunoreactivity in the suburothelium and detrusor layer, no labelling within urothelium or muscle cells.
[103]	Human	Urothelial cells and suburothelial tissues.	Human bladders of OAB patients received Lipotoxin (liposome encapsulated BoNT/A). IHC performed at baseline and 3 months post treatment.	SNAP-25 was expressed in the urothelium and suburothelial nerve fibers, expression decreased post-treatment in patients responding to treatment.
[110]	Human	Bladder mucosa (including the urothelium, lamina propria and a few discontinuous muscularis mucosa).	Human bladders of OAB patients received Lipotoxin and BoNT/A injection. IHC and immunoblotting performed at baseline and 3 months post-treatment.	SNAP-25 expressed in the bladder mucosa, expression was significantly reduced in patients who received BoNT/A injection, suggesting cleavage, but not in patients who received Lipotoxin.
[111]	Human	Intradetrusor nerve fibres.	Human bladders of NDO patients received BoNT/A injection and samples were taken for IHC. However, they did not mention the composition of the samples (urothelium, lamina propria or detrusor).	SNAP-25 expression was shown by IF in the neuronal fibers within the detrusor smooth muscle in untreated and treated patients.
[112]	Rat	Urothelium, suburothelium and muscle (images not clear as to whether expression is in the muscle cells or nerve fibers).	Treated rat bladders with BoNT/A using different protocols, conducted IHC for SNAP-25 on samples taken post-treatment.	SNAP-25 expression was shown by IHC in the bladder; however, it is unclear which cell types specifically. BoNT/A injection significantly reduced SNAP-25 expression, suggesting cleavage.
[102]	Rat	Suburothelial nerve fibers.	Treated rat bladders with Lipotoxin or BoNT/A instillation, conducted IF and western blotting for SNAP-25.	Liposome only and BoNT/A instilled bladders showed staining for SNAP-25 in the subuthelial nerve fibers, however Lipotoxin treated bladders showed significantly reduced SNAP-25 staining, suggesting cleavage.
[113]	Rat	Bladder (does not specify where in the bladder expression was found).	Treated rat bladders with BoNT/A after inducing CYP model of interstitial cystitis. Conducted IHC or immunoblotting for SNAP-25.	SNAP-25 expression was significantly reduced after BoNT treatment, suggesting cleavage.
[99]	Rat	No expression in the urothelium.	Investigated SNARE complexes in urothelium, conducted IF, electron microscopy and western blotting.	Mouse urothelium expressed SNAP-23, SNAP-25 was not found in the urothelium, through IF or Western blot, however was present in choroid plexus (brain stem) tissue that was used as control.
[98]	Mouse	No expression in the urothelium.	Investigated SNARE complexes in the urothelium and bladder, conducted immunoblotting for t-SNAREs and v-SNAREs.	Mouse urothelium expresses SNAP-23 but not SNAP-25.
[114]	Rat	Intradetrusor fibres and cultured DRG.	Injected rat bladders with BoNT/A, performed IHC Cultured DRG cells were treated with BoNT/A and performed ICC.	SNAP-25 expression found in intradetrusor fibers and cultured DRGs.

**Table 3:** Summary of studies that have investigated expression of cleaved SNAP-25 in the bladder after treatment with BoNT/A.

Reference	Species	Where Was Cleaved SNAP-25 Detected?	Methods	Findings
[104]	Guinea pig	Suburothelial nerve fibers only.	Guinea pig bladders were treated with BoNT/A, through intravesical instillation and intramural injection, conducted IHC on bladders for cleaved SNAP-25.	Cleaved SNAP-25 immunoreactive fibers in the mucosa and muscular layer. BoNT/A instillation did not cleave SNAP-25.
[111]	Human	Intradetrusor nerve fibers.	Patients with neurogenic detrusor overactivity (NDO) received intradetrusor BoNT/A. Western blot and immunofluorescence for cleaved SNAP-25 expression.	Detected cleaved SNAP-25 in bladder samples using Western blotting.
[115]	Mouse	Suburothelial nerve fibers.	Injected 0.5U of Botox or Dysport into dome of mouse bladders, three days later conducted IHC of cleaved SNAP-25.	Injection of both forms of BoNT/A led to cleavage of SNAP-25 in nerve fibers of the lamina propria. However, images show the whole bladder, and zoom in only on the lamina propria. Appears to be some staining in the urothelium of the Botox-treated bladder.
[110]	Human	Bladder mucosa (including the urothelium, lamina propria and a few discontinuous muscularis mucosa).	Human bladders of OAB patients received Lipotoxin and BoNT/A injection. IHC and immunoblotting performed at baseline and 3 months post-treatment.	Cleaved SNAP-25 was found in bladder mucosa of patients who received BoNT/A injection, not Lipotoxin.
[114]	Rat	Intradetrusor fibres and cultured DRGs.	Injected rat bladders with BoNT/A, performed IHC Cultured DRG cells were treated with BoNT/A and performed ICC.	Cleaved SNAP-25 was found in intradetrusor fibers and cultured DRGs.

A summary of studies reporting SV2 expression is provided in Table 4. Expression has been shown in rat and human urothelial cells [41,116], while other studies report it is only expressed in the suburothelial or intradetrusor nerve fibers [101,110,117,118,119] with denser immunostaining in parasympathetic nerves than sympathetic or sensory nerves [101]. Interestingly, there have been no studies showing the presence of SV2 and SNAP-25 in detrusor smooth muscle cells, most reports show expression is restricted to nerve fibers running through the detrusor [101,104,117].

There is evidence to suggest BoNT/A could enter urothelial cells through a non-SV2 mediated mechanism. Jacky et al. (2013) identified fibroblast growth factor receptor 3 (FGFR3) as a receptor for BoNT/A and showed that higher FGFR3 expression in neuronal cells led to increased toxin internalization [120]. Bomba-Warczak et al. (2016) also reported increased cleavage of SNAP-25 in neurons after expression of FGFR3 [121]. This receptor could potentially facilitate BoNT/A uptake in the urothelium as it has been shown that FGFR3 is expressed in the urothelium and mutations in the FGFR3 gene contribute to the development of urothelial carcinoma [122].

**Table 4:** Summary of studies that have investigated expression of SV2 in the bladder.

Reference	Species	Where Was SV2 Detected?	Methods	Findings
[117]	Human	Dorsal root ganglion neurons and nerve fibers within the bladder.	Collected bladder tissue from patients with IDO, PBS and controls, cultured human DRG neurons, Conducted IHC on bladder tissue and calcium imaging on DRG neurons.	SV2 was expressed in DRG neurons, immunoreactivity significantly increased in injured neurons. SV2 expressed in nerve fibers within the urothelium, suburothelium, and detrusor.
[116]	Human	Urothelial cells.	Cultured human urothelial cell lines and conducted PCR.	Urothelial cells express SV2-A and SV2-B.
[118]	Human	Parasympathetic nerves innervating detrusor.	Biopsies of detrusor muscle from patients with sensory urgency and control patients, conducted IHC for SV2 and P2X receptors.	Used SV2 as a neuronal marker for parasympathetic nerves. There was no staining in detrusor muscle itself.
[103]	Human	Urothelium and suburothelial fibers.	Instilled 200U Lipotoxin (liposome encapsulated BoNT/A) into bladders of OAB patients, IHC for SV2 expression before treatment and 3 months after.	IHC and western blotting shows SV2 expression in the urothelium (apical cells and suburothelium).
[101]	Human	Nerve fibers in the suburothelium and detrusor.	Human bladders from organ donors were used for immunofluorescence staining of SV2.	Dense SV2 immunoreactivity in the suburothelium and detrusor layer colocalized with VAChT and CGRP positive fibers. They found no labelling within urothelium or muscle cells.
[41]	Rat and human	Bladder mucosa.	IHC and gel electrophoresis of rat and human bladder mucosa (urothelium and lamina propria) and cultured urothelial cells.	SV2 expression found in human and rat mucosa, and rat cultured urothelial cells. Expression was not found in human urothelial cells.
[110]	Human	Bladder mucosa (including the urothelium, lamina propria and a few discontinuous muscularis mucosa).	Human bladders of OAB patients received Lipotoxin and BoNT/A injection. IHC and immunoblotting performed at baseline and 3 months post treatment.	SV2 expression found in mucosa of OAB patients at baseline and one month after BoNT/A injection and Lipotoxin treatment.
[123]	Guinea pig	Suburothelial nerve fibers.	IHC staining for SV2 conducted on fixed guinea pig bladders.	SV2 expression found in suburothelial nerve fibers, authors used SV2 as a marker for efferent nerves.

The different findings between various studies reporting expression patterns of SV2 and SNAP-25 could be due to myriad reasons. Species differences do not seem sufficient to account for the different findings, as the tables above show studies that have been performed on human, rat, guinea pig, and mouse tissue reported conflicting results. Cleaved SNAP-25-specific staining has been used to demonstrate function of BoNT/A (Table 3); this approach (staining for cleaved but not total SNAP-25) only reveals the totality of whether BoNT/A has intoxicated cells and cleaved SNAP-25 in the sample, it does not reveal if SNAP-25 may be present but somehow protected from the BoNT/A, as BoNT/A can only cleave proteins it can access within intoxicated cells. Different routes of administration of BoNT/A, might easily affect target accessibility. Furthermore, when investigating the presence of SNAP-25 in the urothelium, some investigators have performed western blots using the bladder ‘mucosa’, possibly including the suburothelium which contains both efferent and afferent nerve fibers and interstitial cells. These studies are not therefore incompatible with the findings of studies that report SNAP-25 is only present in the suburothelium. There are also the issues of selectivity and sensitivity of antibodies, as potential false positive labeling is a well-known disadvantage of immunological methods [124]. Perhaps the use of complementary and more quantitative methods or knock out mouse models could help to dispel the remaining confusion.

## 9. Evidence in Support of a Classical Mechanism for BoNT/A in the Bladder

Although there is evidence to suggest BoNT/A modulates bladder function through inhibition of sensory neurotransmission, there is also evidence pointing to it working through the well-characterized mechanism of inhibition of efferent cholinergic signaling. It is unlikely BoNT/A would affect urothelial release of ACh, which has been shown to occur through a non-vesicular mechanism [93]; however, the suburothelial and intramuscular efferent nerves are essential to the mediation of bladder contraction. As SNAP-25 is part of a vesicular fusion complex that mediates release of ACh-containing vesicles, its cleavage inhibits the function of this complex and effectively silences the neuron and in turn paralyses the muscle [5].

Preclinical studies have shown that BoNT/A inhibits electric field stimulation (EFS) mediated bladder contraction in rodent models [125,126,127,128,129,130]. BoNT/A injections into rat bladders led to a significant decrease in EFS evoked ACh release 5 days after treatment. Smith et al. (2003) and Lawrence et al. (2010) both reported that BoNT/A inhibited EFS-induced cholinergic and purinergic-mediated contractions in rat bladder strip assays [125,130]. When taken together, this all suggests that BoNT/A directly silences cholinergic nerves. However, there are also studies reporting that BoNT/A did not inhibit detrusor contractility. Howles et al. (2009) reported that BoNT/A application did not affect guinea pig and mouse detrusor strip and whole bladder contraction [131] and Munoz et al. (2011) found no alterations in voiding contraction amplitude in cystometric analysis of rats with spinal cord injury [39]. One explanation for these discrepancies could be that because BoNT/A is a large molecule, at 150 kDa, it is unlikely to diffuse and move easily between tissues, therefore the effect of BoNT/A treatment may be highly dependent on its penetrance into the bladder wall. In the study by Howels et al. (2009), the BoNT/A was applied in the bathing solution and not directly injected into the tissue, the toxin might therefore not have been able to penetrate the layers of the bladder wall to reach its site of action (i.e., the efferent nerves) a lack of effect on contractility in these studies could be due to differences in concentration and preparation of BoNT/A used as well as incubation time of the compound.

## 10. Use of Animal Models in Urology Research

Over the past few decades, the use of animals in urology research has provided invaluable insights into bladder function in health and disease, as well as aiding development of pharmacological treatments for OAB and IC/PBS. We can measure different urological parameters to dissect the function of a specific pathway and provide evidence to inform clinical studies and eventual treatment recommendations to improve patients’ quality of life.

An effective animal model must meet certain criteria; it must have similar symptomology to the human disorder, it must have similar pathophysiology to the human disorder, and drugs already used to treat patients should also reverse the induced symptoms in the model [132]. The most commonly used animals in research are rats and mice, there are many advantages to using rodents in urology research as it is cost-effective, their physiology is like humans and they have relatively short gestation periods which allows quick generation of knock-out/in models. Pig bladders have been shown to be similar in physiology to human bladders [133], and studies using porcine tissue have provided insight to bladder function [78,134,135].

The fact that micturition in humans is not simply based on the level of fullness of the bladder, but also social factors is an added complication of modeling urological disorders in animals as it represents a major difference between human and animal physiology. We are fundamentally unable to model OAB accurately in animals as its cardinal symptom is urgency, which is subjective and requires communication with the patient. This led to the need for urodynamic assessment of healthy human subjects. Wyndaele and De Wachter (2002) conducted a cystometric study with 50 young healthy volunteers and described the three main sensations associated with bladder filling; the first sensation of bladder filling, the first desire to void and the strong desire to void [136]. The International Continence Society (ICS) uses these parameters to define urgency for the diagnosis of OAB [15]. Interestingly, BoNT/A has been shown to significantly increase the volume of first desire to void after injection [10,137], which is evidence of its sensory effects.

Many studies have analyzed voiding behavior of animals using methods such as metabolic cages and voiding spot assays; however, rodents are nocturnal and as they drink more often in nighttime, they also urinate more often which should be kept in mind when conducting urodynamic assessments [138]. There are differences in physiology which could affect translatability of findings from rodent studies. For example, the activation of healthy human detrusor smooth muscle is mediated almost entirely by ACh, whereas rodent detrusor tissue is activated by a combination of ACh and ATP signaling [25]. Interestingly, purinergic driven contraction has been shown in patients with LUT disorders as some contraction persists in the presence of atropine which blocks ACh receptors, suggesting that increased ATP release occurs in bladder dysfunction which is not present in the healthy bladder [25,139]. This could suggest the normal rodent bladder is a good model for human bladder disorders.

## 11. BoNT Research and Future Prospects

As discussed above, there are many gaps in the understanding of BoNT/A-mediated inhibition of sensory nerve function, and further research could improve the safety profile and specificity of BoNT/A. Although millions of patients worldwide have received BoNT/A injection, clinical or cosmetic, it remains the deadliest natural neurotoxin [140]. The remarkable selectivity of BoNT/A allows it to be exploited to silence specific neuronal populations.

A lot of work has gone into innovating ways to improve penetrance of BoNT/A into human bladders, to reduce the risk of deleterious effects such as urinary retention and provide less invasive methods of treatment, including liposomes [103,119], low-energy shock waves [141] and inert hydrogels [142]. Liposomes have been shown to improve bladder function after intravesical administration in rats with acetic acid induced bladder hyperactivity, potentially by restoring barrier function of the urothelium to stop noxious substances reaching the suburothelial afferent fibers [143]. Intravesical liposomes have been shown to be effective in patients with IC/PBS, significantly reducing pain and urgency [144]. Loading liposomes with BoNT/A could have the dual benefit of improving barrier function and delivering BoNT/A into the bladder through a non-invasive method, animal and clinical studies have shown improvements in bladder function post Lipotoxin treatment [102,103,107,110,145]. Chuang and Kuo (2017) found improvements in IC/PBS symptoms after treatment however they attributed this to a placebo effect [146]. Further clinical studies are needed to better understand the function of Lipotoxin in improving symptoms of LUT disorders.

## 12. Conclusions

In summary, BoNT/A has been proven to be safe and effective in treating LUT disorders; however, we are still not completely aware of how it acts, at a molecular level, to improve sensory symptoms. The lack of consensus over the presence of BoNT/A’s receptor and target in bladder tissue prompts the need for further research to improve our general understanding. Developing BoNT treatment for OAB and IC/PBS could include engineering of recombinant toxins specific to the bladder or expanding delivery methods that allow BoNT/A to enter into the bladder wall. This could lead to the inhibition of sensory neurotransmission long-term while avoiding invasive and scarring injections to provide lasting relief for patients with LUT disorders.

## 13. Limitations

Limitations of this review included use of only one search database (Pubmed), although every endeavor was made to include all relevant papers, some may have been missed.

## 14. Methods

A comprehensive search of the Pubmed database was conducted to identify English-language, original research articles reporting clinical or preclinical data on effect of Botulinum neurotoxin serotype A (BoNT/A) on bladder sensation. The search terms used are shown below.

“botulinum neurotoxin”. “BoNT/A”, “bladder”, “urothelium”, “afferent”, “sensory”, “SNAP-25”, “synaptosomal associated protein 25”, “SV2”, “synaptic protein 2”, “overactive bladder”, “OAB”, “interstitial cystitis/bladder pain syndrome”, “IC/BPS”, “receptor” and “exocytosis”.

These terms were searched with Boolean terms (AND/OR) to refine results.

Duplicate results were removed, and articles were screened for eligibility. Review articles were not included. Initial screening included assessment of titles and abstracts for relevance. Secondary screening of full-text articles was conducted on relevant studies. There were no limits on date of publication, or study design. Following the full-text screening, the references of key publications were searched for possible additions to the study. Pertinent data were extracted and presented in table form.

**Figure 2:**
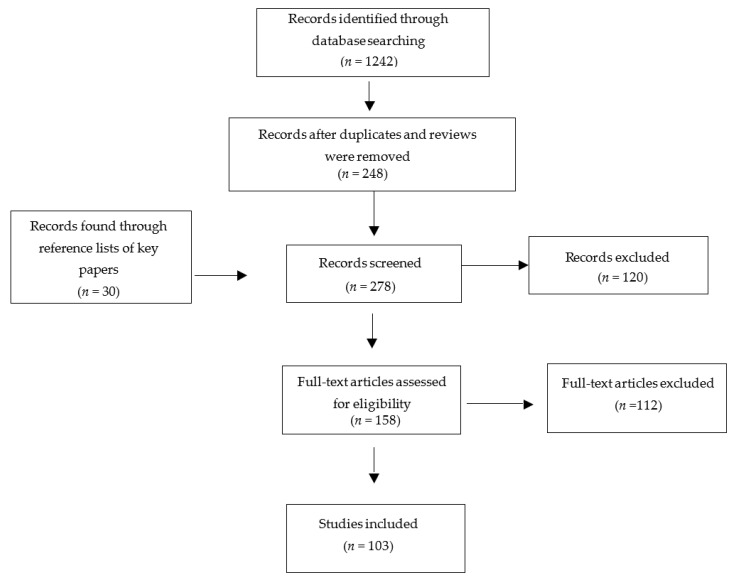
Results from the search strategy.

A total of 1242 results were obtained initially, further searches were then conducted to exclude duplicates, reviews and clinical trials. Of the abstracts screened, 248 were deemed relevant and taken forward to full-text screening (Figure 2). The reference lists of key articles were searched, and relevant articles were also screened. Of the full-text articles screened, 103 were included. Data were extracted from selected articles, 11 articles provided data for the expression of total SNAP-25, five articles provided data for cleaved SNAP-25 and eight articles provided data for SV2 expression (Table 2, Table 3 and Table 4).

## Figures and Tables

**Table 1:** Abbreviations of bladder disorders and their definitions (Adapted with permission from ref. [15]. Copyright 2003 Elsevier Inc.).

Bladder Disorder		Definition
OAB	Overactive bladder	A syndrome characterized by urinary urgency (the sudden need to urinate that cannot be deferred to later), frequency (needing to urinate more often), and nocturia (sleep disturbances caused by increased need to urinate) which can be accompanied by incontinence (inability to hold your bladder).
NDO	Neurogenic detrusor overactivity	Involuntary contraction of the smooth muscle of the bladder (the detrusor) during the storage phase. Defined as neurogenic when it occurs due to a neurological condition such as spinal cord injury.
IDO	Idiopathic detrusor overactivity	Involuntary contraction of the detrusor with no known cause.
IC/PBS	Interstitial cystitis/painful bladder syndrome	Pain that accompanies bladder filling, which may lead to frequency and nocturia. For the diagnosis of interstitial cystitis, cystoscopy and histological assessments are necessary.
BOO	Bladder outlet obstruction	When an obstruction in the urethra (most commonly an enlarged prostate) makes voiding difficult and can increase detrusor pressure due to the increased residual volume in the bladder.

## Data Availability

No new data were created or analyzed in this study. Data sharing is not applicable to this article.
